# Chorioamnionitis, neuroinflammation, and injury: timing is key in the preterm ovine fetus

**DOI:** 10.1186/s12974-018-1149-x

**Published:** 2018-04-19

**Authors:** Ruth Gussenhoven, Rob J. J. Westerlaken, Daan R. M. G. Ophelders, Alan H. Jobe, Matthew W. Kemp, Suhas G. Kallapur, Luc J. Zimmermann, Per T. Sangild, Stanislava Pankratova, Pierre Gressens, Boris W. Kramer, Bobbi Fleiss, Tim G. A. M. Wolfs

**Affiliations:** 1 Department of Pediatrics, Maastricht University Medical Center, 6202 AZ Maastricht, The Netherlands; 2 School for Mental Health and Neuroscience (MHeNs), Maastricht University Medical Center, 6229 ER Maastricht, The Netherlands; 3 School of Oncology and Developmental Biology (GROW), Maastricht University Medical Center, 6229 ER Maastricht, the Netherlands; 4 Division of Neonatology/Pulmonary Biology, The Perinatal Institute, Cincinnati Children’s Hospital Medical Center, University of Cincinnati, Cincinnati, OH 45208 USA; 5 School of Women’s and Infants’ Health, The University of Western Australia (M550), Crawley, WA 6009 Australia; 6 Department of Comparative Pediatrics and Nutrition, Faculty of Health and Medical Sciences, University of Copenhagen, Frederiksberg DK 1870 C, Copenhagen, Denmark; 7 Departments of Pediatrics and Adolescent Medicine, Rigshospitalet, Copenhagen, 2100 Denmark; 8 Department of Perinatal Imaging and Health, Department of Division of Imaging Sciences and Biomedical Engineering, King’s College London, King’s Health Partners, St. Thomas Hospital, London, SE1 7EH UK; 9 PROTECT, INSERM, Université Paris Diderot, Sorbonne Paris Cité, Paris, France; 10 PremUP, Université Paris Diderot, Sorbonne Paris Cite, Paris, France; 11 Department of BioMedical Engineering, Maastricht University Medical Center, 6229 ER Maastricht, The Netherlands

**Keywords:** Chorioamnionitis, Fetal, Preterm, Sheep, Inflammation, Brain injury, Erythropoietin, EPO receptor

## Abstract

**Background:**

Antenatal infection (i.e., chorioamnionitis) is an important risk factor for adverse neurodevelopmental outcomes after preterm birth. Destructive and developmental disturbances of the white matter are hallmarks of preterm brain injury. Understanding the temporal effects of antenatal infection in relation to the onset of neurological injury is crucial for the development of neurotherapeutics for preterm infants. However, these dynamics remain unstudied.

**Methods:**

Time-mated ewes were intra-amniotically injected with lipopolysaccharide at 5, 12, or 24 h or 2, 4, 8, or 15 days before preterm delivery at 125 days gestational age (term ~ 150 days). Post mortem analyses for peripheral immune activation, neuroinflammation, and white matter/neuronal injury were performed. Moreover, considering the neuroprotective potential of erythropoietin (EPO) for perinatal brain injury, we evaluated (phosphorylated) EPO receptor (pEPOR) expression in the fetal brain following LPS exposure.

**Results:**

Intra-amniotic exposure to this single bolus of LPS resulted in a biphasic systemic IL-6 and IL-8 response. In the developing brain, intra-amniotic LPS exposure induces a persistent microgliosis (IBA-1 immunoreactivity) but a shorter-lived increase in the pro-inflammatory marker COX-2. Cell death (caspase-3 immunoreactivity) was only observed when LPS exposure was greater than 8 days in the white matter, and there was a reduction in the number of (pre) oligodendrocytes (Olig2- and PDGFRα-positive cells) within the white matter at 15 days post LPS exposure only. pEPOR expression displayed a striking biphasic regulation following LPS exposure which may help explain contradicting results among clinical trials that tested EPO for the prevention of preterm brain injury.

**Conclusion:**

We provide increased understanding of the spatiotemporal pathophysiological changes in the preterm brain following intra-amniotic inflammation which may aid development of new interventions or implement interventions more effectively to prevent perinatal brain damage.

## Background

Antenatal infections (i.e., chorioamnionitis) are an important risk factor for preterm birth and a major contributor to neonatal morbidity and mortality [1, 2]. Intra-amniotic exposure to microorganisms and subsequent induction of inflammatory mediators in the amniotic cavity can initiate a fetal systemic immune response that is characterized by increased plasma interleukin (IL)-6 and IL-8 concentrations [3], and (persistent) changes in essential immunological organs including the fetal spleen and thymus [4, 5]. At the crosstalk between fetal peripheral blood and the brain (i.e., blood-brain barrier), this systemic inflammatory response can initiate a detrimental neuroinflammatory response which is primarily mediated by microglia and peripheral immune effector cells [6, 5]. This cerebral inflammatory response is a risk factor for preterm brain injury and concomitant adverse neurodevelopmental outcomes including cognitive, behavioral, and attentional impairments and motor dysfunctions (i.e., cerebral palsy) [7, 5].

In a pre-clinical chorioamnionitis model, we showed that short-term (2 days) intra-amniotic exposure to lipopolysaccharide (LPS) resulted in systemic inflammation, overt microgliosis, and changes in myelin basic protein (MBP) immunoreactivity (IR) in the fetal ovine brain [8]. However, this systemic and cerebral phenotype was substantially different following longer exposure time (7 days) indicating that time-dependent peripheral and cerebral changes occur following intra-amniotic inflammation. Moreover, we and others have shown that inflammation can modulate a second inflammatory stimulus through either preconditioning or sensitization of the fetal brain [9, 8, 10]. Taken together, this emphasizes that inflammation as pathogenic mediator for brain damage is not a single trigger within a short time frame but more a dynamic process over an extended period of time. Therefore, detailed studies elucidating the time-dependent effects of antenatal infection/inflammation in relation to neurological injury and development are crucial to gain insight in the pathophysiological changes in the fetal brain following antenatal stress. Importantly, such temporal insight in the induction of brain injury following antenatal stress is also essential to define the therapeutic window of opportunity for neurotherapeutics.

One of the most promising treatment options for preterm neonates at high risk for brain injury is erythropoietin (EPO), an important cytokine for brain development [11, 12]. Multiple experimental and clinical studies have demonstrated efficacy of EPO administration to prevent injury to the preterm brain, including severe periventricular leukomalacia, without adverse effects [13–21]. In contrast, other clinical trials do not report improvement in neurodevelopment following EPO treatment [22, 23]. Effects of EPO are mediated by its receptor, which is abundantly present on (pre) oligodendrocytes, astrocytes, microglia, and neurons. EPO binding triggers phosphorylation of two monomers, which in turn phosphorylates and activates the signaling kinase Jak-2 facilitating effects including its anti-inflammatory, anti-oxidative, and anti-apoptotic properties [24, 25]. In addition, EPO enhances neuro- and oligodendrogenesis, oligodendrocyte maturation, and myelin production which are indispensable events in injury repair and normal neurodevelopment [26]. We hypothesize that changes in basal levels of EPO receptor activation in response to inflammation or perinatal stress might explain at least part of the differences in clinical outcomes following EPO treatment.

Considering the clinical need for understanding the time-dependent cerebral changes following intra-amniotic inflammation, we performed a detailed analysis of the temporal dynamics of intra-amniotic LPS-induced systemic and cerebral inflammation and subsequent fetal brain injury. In addition, to optimize EPO treatment in the clinical setting, we analyzed the temporal expression of the phosphorylated EPO receptor (pEPOR) in the course of intra-amniotic inflammation.

## Methods

### Study approval

Animal procedures were performed with approval of the animal ethics committee of the University of Western Australia (Perth, Australia).

### Experimental design

**Fig. 1:**
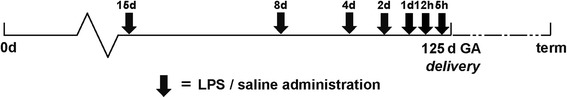
Study design. Pregnant ewes received an intra-amniotic injection with 10 mg Escherichia coli-derived lipopolysaccharide (LPS) at 5, 12, or 24 h or 2, 4, 8, or 15 days (black arrows) before preterm delivery at 122 days of gestation (term ~ 150 days). Control animals received an intra-amniotic injection with an equivalent volume of 0.9% saline solution at comparable time points to LPS injections

The design of this study was published previously [4]. Briefly, 52 time-mated ewes with singleton fetuses were randomly allocated in groups of 5–7 animals per group to receive an intra-amniotic injection under ultrasound guidance with an established dose [27] of 10 mg *Escherichia coli*-derived LPS (O55:B5; Sigma-Aldrich, St. Louis, MO) at 5, 12, or 24 h or 2, 4, 8, or 15 days before preterm delivery at 125 days of gestation (term ~ 150 days) (Fig. 1). This paradigm is based on the clinical paradigm, where we know the gestational age of the infant but not the length of exposure to inflammation. As such, all our tissues were collected at a known gestation age but inflammation was induced at various times before. The half-life time of LPS in the amniotic fluid is relatively long (1.7 days) and LPS concentrations remain detectable till 15 days after injection [28]. Moreover, intra-amniotic delivery of 0.1 mg LPS, a bolus which in this study is reached at 10 days after injection, still results in an influx of inflammatory cells in the amniotic fluid and fetal lungs [27] indicating that IA delivery of 10 mg LPS is a clinical relevant ongoing inflammatory stimulus. Fetuses of either sex were used, and previous analysis of the thymus reported no sex specific differences in this model [4]. Control animals received an equivalent volume of 0.9% saline solution (SAL; controls) at variable gestational ages comparable to LPS injections, ranging from 5 h to 15 days before preterm delivery. Within this control group, no differences were observed between different lengths of saline exposure for which we have pooled these animals in one control group (SAL). At 125 days of gestation, when ovine brain development is similar to 32–34 weeks of human gestation [29], all fetuses were surgically delivered and immediately euthanized with intravenous pentobarbitone (100 mg/kg). Fetal blood was collected and the brains were removed and immersion fixed in 4% paraformaldehyde.

### Analysis of blood IL-6 and IL-8 concentration

Levels of the pro-inflammatory cytokines interleukin (IL)-6 and IL-8 were measured in fetal plasma as markers for systemic inflammation using ovine-specific sandwich enzyme-linked immunosorbent assays (ELISA) as previously described [8].

In short, a 96-wells plate was coated with a monoclonal mouse-anti IL-6 (Millipore Cat# MAB1004, working concentration 1:200) or IL-8 (Millipore Cat# MAB1044, working concentration 1:200) and incubated overnight at 4 °C. The standard curve and serum samples were diluted in PBS + 0.1% BSA in 1:1 or 1:80, respectively, for IL-6 and IL-8. Incubation with the detection antibody rabbit-anti-ovine IL-6 (Millipore Cat# AB1839, working concentration 1:500) or IL-8 (AB1040, Millipore, working concentration 1:500) was performed for 1 h, followed by incubation with a HRP-labeled antibody (Jackson ImmunoResearch Labs Cat# 111-035-045, working concentration 1:500). Next, incubation with 3,3′5,5′-tetramethylbenzidine (TMB) substrate solution was done for 10 (IL-6) or 2,5 (IL-8) minutes. The reaction was stopped by addition of H_2_SO_4_, and the optical density (OD) was measured at 450 nm in a Thermo Electron Type 1500 Multiskan Spectrum Microplate Reader. Concentrations were expressed relative to a standard curve of recombinant ovine IL-6 or IL-8 (ImmunoChemistry Technologies, Bloomington, MN, USA).

### Histology and immunohistochemistry

The cerebral white matter and hippocampus are most commonly affected by intra-amniotic infections at this developmental stage [30]. Therefore, we have chosen to assess inflammatory and structural changes within these regions of interest. After fixation, a predefined region containing the posterior hippocampus/mid-thalamus of the left hemisphere was embedded in paraffin and serial coronal sections (4 μm) were cut with a Leica RM2235 microtome. Hematoxylin and eosin (H&E) staining was performed for structural and morphological analysis. Immunohistochemical staining was performed on four slides per staining per animal (every 10th consecutive slide) as previously reported [8]. Inflammatory changes were assessed by the following immunohistochemical markers: cylcooxygenase-2 (COX-2) (1:50, Cayman Chemical; aa570-598), ionized calcium-binding adapter molecule 1 (IBA-1) (1:1000, Wako Pure Chemical Industries, Osaka, Japan), and glial fibrillary acidic protein (GFAP) (1:1000, DAKO Z0334). The presence of neutrophils was assessed by myeloperoxidase (MPO) staining (1:200, DAKO A0398). Markers used to assess alterations in the white matter including oligodendrocyte differentiation were oligodendrocyte transcription factor 2 (Olig2) (1:200, Millipore, 13 AB9610), platelet-derived growth factor receptor alpha (PDGFRa) (1:100, Santa Cruz Biotechnology, sc338), 2′,3′-cyclic-nucleotide 3′-phosphodiesterase (CNPase) (1:1000, Sigma, C5922), and myelin basic protein (MBP) (1:1000, Merck Millipore, MAB386). Neuronal architecture, including cell bodies and dendrites, was determined by microtubule-associated protein-2 (MAP-2) (1:500, Sigma, M9942). Apoptotic cell death was measured as cells positive for cleaved caspase-3 (1:1000, cell signaling, #9661), and the number of mitotic cells were identified by phospho-Histone H3 (pHH3) (1:100, Santa Cruz Biotechnology, sc-101,679). The presence of the erythropoietin receptor was assessed by measuring the expression of the (phosphorylated) erythropoietin receptor (EPOR and pEPOR) (1:200, Santa Cruz, SC-365662 and SC-20236).

Deparaffinization and rehydration was performed by incubation in xylol and decreasing alcohol concentrations. Endogenous peroxidase activity was quenched via incubation with 0.3% H_2_O_2_ for 10 min. Antigen retrieval involved boiling tissues in citrate buffer (pH 6.0) for 10 min or for pEPOR proteinase K at 37 °C for 5 min. Nonspecific binding was prevented by incubation with 5% (IBA-1, GFAP, MAP2, pHH3) or 10% (MPO, CNPase) normal goat serum, 5% bovine serum albumin (COX-2, MBP, Olig2, EPOR) (Invitrogen Thermofisher Scientific), or 10% nonfat dry milk (pEPOR; Elk, Campina bv., Eindhoven, The Netherlands) for 1 h. Tissues were incubated with the primary antibody overnight at 4 °C, followed by incubation with the species specific secondary antibody at 1:200 (DAKO) for 1 h at room temperature. The antibody-specific signal was enhanced with a Vectastain ABC peroxidase Elite kit (Vector Laboratories Inc., Burlingame, CA) for 1 h and 3,3′-diaminobenzide (COX-2, IBA-1, GFAP, MPO, PDGFRa, CNPase, MBP, MAP-2, pHH3, EPOR) or nickel chloride 3,3′-diaminobenzide (Olig2, cleaved caspase-3, pEPOR) for 2–10 min. Nuclei were stained with Mayer’s hematoxylin.

### Qualitative and quantitative analysis

An independent neuropathologist and two independent researchers who were blinded for the experimental conditions performed qualitative and quantitative analysis of the tissues. Analysis was performed using a light microscope (Leica DM2000) equipped with Leica QWin Pro version 3.4.0 software (Leica Microsystems, Mannheim, Germany). H&E-stained sections were scored for gliosis, hemorrhages, and structural damage-like cyst formation. Regions of interest of the white matter and hippocampus were defined as previously described [31]. In addition, gray matter alterations in the cerebral cortex were assessed within the same section. Three to five adjacent images were taken per region of interest at × 100 magnification, and analyses were performed using Leica Qwin Pro v3.4.0. software (Leica Microsystems, Wetzlar, Germany). Area fractions and integrated densities were calculated for IBA-1, GFAP, COX-2, MBP, MAP-2, and pEPOR. MPO, Olig2, PDGFRa, CNPase, pHH3, and cleaved caspase-3-positive cells were counted and expressed as total cell count per square millimeter (cells/mm^2^). In addition, MPO+ cells were also counted in the choroid plexus. Values per region of interest were averaged.

### Statistical analysis

All values are shown as mean with 95% confidence interval (CI) or standard deviations (SD). Comparison between different experimental groups was performed with analysis of variance (ANOVA) or with a random intercept-mixed model in case of repeated measurements per animal (e.g., different sections per brain) with Bonferroni correction for multiple comparisons. We applied log transformation to obtain normal distributed data when data or variables were positively skewed before statistical testing. Statistical analysis was performed with IBM SPSS Statistics Version 22.0 (IBM Corp., Armonk, NY, USA; SPSS). Statistical significance was accepted at *p* < 0.05. Considering the relatively low number of animals per group, exact *p* values are provided and 0.05 < *p* < 0.1 is considered a trend.

## Results

### Animal characteristics

At birth, no differences in weight were found between experimental groups. Fetal blood pH and hemoglobin levels did not differ following intra-amniotic LPS exposure. No sex differences in susceptibility were observed in either readout including animal characteristics and all following readouts regarding systemic cytokine levels and immunohistochemical analysis.

### Intra-amniotic LPS exposure results in a biphasic fetal systemic inflammatory response

**Fig. 2:**
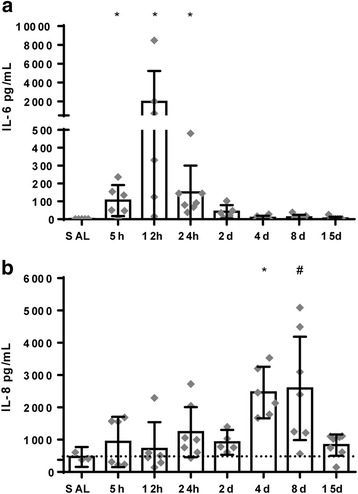
**a**–**b** Circulatory interleukin (IL)-6 and IL-8 concentrations illustrate a biphasic response following intra-amniotic LPS exposure. Undetectable values were assigned an arbitrary value of 1 pg/mL in order to perform statistical analysis. Statistical analysis was done with ANOVA, and values are expressed as mean ± 95% CI. Asterisk indicated *p* < 0.05 versus control group; number sign indicated 0.05 < *p* < 0.1 versus control

Intra-amniotic exposure to LPS results in an acute increase of fetal systemic IL-6 concentrations at 5, 12, and 24 h after LPS exposure compared to control levels (SAL vs 5 h LPS *p* = 0.0157; SAL vs 12 h LPS *p* = 0.0011; SAL vs 24 h LPS *p* = 0.0035) (Fig. 2a). Subsequently, at 4 and 8 days after LPS exposure, systemic IL-8 concentrations are increased compared to controls (SAL vs. 4 days LPS *p* = 0.0147; SAL vs. 8 days LPS *p* = 0.0502) (Fig. 2b).

### Cerebral inflammation in the fetal white matter, hippocampus, and cortex following intra-amniotic LPS exposure

**Fig. 3:**
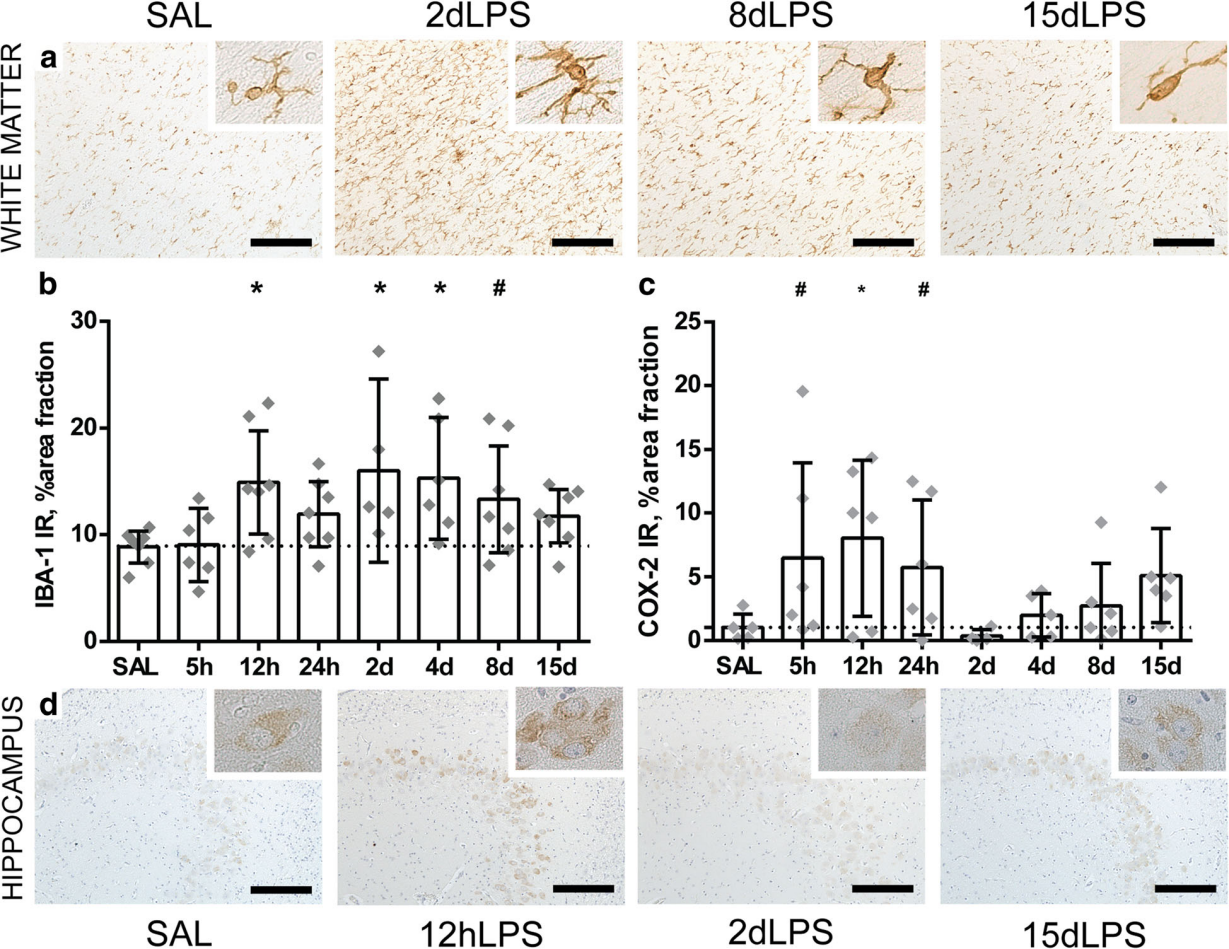
Intra-amniotic exposure to LPS induces an acute, transient cerebral inflammatory response in the preterm white matter and hippocampus. An increase of the area fraction of IBA-1 immunoreactivity (IR) was observed in the white matter at 12 h, 2 days, 4 days, and 8 days following LPS exposure compared to controls (SAL vs. 12 h LPS *p* = 0.012; SAL vs. 2 days LPS *p* = 0.006; SAL vs. 4 days LPS *p* = 0.005; SAL vs. 8 days LPS *p* = 0.088) (**a**, **b**). In the hippocampus, an increase of the area fraction of COX-2 IR was found at 5, 12, and 24 h following LPS exposure (SAL vs. 5 h LPS *p* = 0.055; SAL vs. 12 h LPS *p* = 0.016; SAL vs. 24 h LPS *p* = 0.096) (**c**, **d**). Representative histological figures of the IBA-1-positive microglia in animals exposed to intra-amniotic saline (SAL), 2, 8, and 15 days of LPS are shown in **a**. Representative histological figures of COX-2-positive neurons in the hippocampus of animals exposed to saline (SAL), 12 h, 2 days, and 15 days LPS are depicted in (**d**). IBA-1 IR and COX-2 IR are depicted as mean % area fraction ± 95% CI. Asterisk indicated *p* < 0.05 versus control (SAL); number sign indicated 0.05 < *p* < 0.1 versus control (SAL). Images taken at × 100 magnification (insert at × 400 magnification), scale bar = 200 μm

A systemic fetal inflammatory response is postulated to initiate cerebral inflammation leading to subsequent injury [32–34]. Therefore, we initially measured markers of changes in neuroinflammatory processes (IBA-1, COX-2, GFAP). We found that the systemic inflammatory response following intra-amniotic LPS exposure is followed by cerebral inflammatory changes as indicated by an increase in IBA-1 IR in the white matter at 12 h, 2 days, 4 days, and 8 days following LPS exposure compared to controls (SAL vs. 12 h LPS *p* = 0.012; SAL vs. 2 days LPS *p* = 0.006; SAL vs. 4 days LPS *p* = 0.005; SAL vs. 8 days LPS *p* = 0.088) (Fig. 3a, b). In the hippocampus, inflammation is detected by an acute increase in COX-2 IR at 5, 12, and 24 h post LPS exposure (SAL vs. 5 h LPS *p* = 0.055; SAL vs. 12 h LPS *p* = 0.016; SAL vs. 24 h LPS *p* = 0.096) (Fig. 3c, d) and an increase in IBA-1 IR at 15 days after LPS exposure compared to controls (SAL vs. 15 days LPS *p* = 0.073). There were no changes of GFAP IR after LPS exposure at any of the time points. As outlined in Table 1, the number of MPO+ cells is increased in the white matter at 15 days post LPS exposure (SAL vs. 15 days LPS *p* = 0,027). In the choroid plexus, no significant differences of MPO+ cells were found between groups (Table 1).

**Table 1:** MPO-positive cells in the choroid plexus and white matter

MPO+ cells/mm^2^	SAL	5 hours LPS	12 hours LPS	24 hours LPS	2 days LPS	4 days LPS	8 days LPS	15 days LPS
Choroid plexus	2.44 ± 3.22	4.34 ± 2.86	3.19 ± 3.44	1.80 ± 1.45	3.88 ± 2.54	6.25 ± 4.25	4.07 ± 2.67	3.51 ± 4.38
White matter	0.47 ± 0.30	3.83 ± 3.82	1.14 ± 0.83	1.95 ± 2.46	1.88 ± 2.70	2.05 ± 1.84	2.67 ± 2.50	4.52 ± 4.22*

Mean values ± standard deviations are represented

*p < 0.05

### Cell death and proliferation in the fetal white matter, hippocampus, and cortex following intra-amniotic LPS exposure

**Fig. 4:**
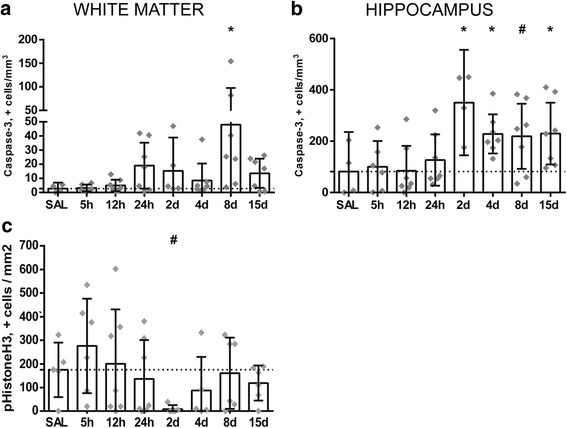
Intra-amniotic exposure to LPS results in a decrease in mitotic cells and relatively late onset of cell death in the preterm white matter and hippocampus. A significant increase of caspase-3-positive cells is observed at 8 days following LPS exposure in the white matter compared to controls (SAL vs 8 days LPS *p* = 0.004) (**a**). In the hippocampus, at 2, 4, 8, and 15 days following LPS exposure an increase in caspase-3-positive cells was found compared to controls (SAL vs 2 days LPS *p* = 0.002; SAL vs 4 days LPS *p* = 0.030; SAL vs 8 days LPS *p* = 0.058; SAL vs 15 days LPS *p* = 0.042) (**b**). At 2 days following LPS exposure, a decrease in pHH3+ cells was found compared to controls (SAL vs. 2 days LPS *p* = 0.100) (**c**). Caspase-3 and pHH3 are expressed as positive cells/mm^2^ and represented in the graphs as mean ± 95% CI. Asterisk indicated *p* < 0.05 versus control (SAL); number sign indicated 0.05 < *p* < 0.1 versus control (SAL)

To assess whether cerebral inflammation is followed by tissue injury, we measured the number of caspase-3-positive cells in the cerebral white matter, hippocampus, and cortex as a marker of apoptotic cell death as this is an important prognostic factor for neurological outcomes [35]. Exposure to LPS results in an increase in cleaved caspase-3-positive cells in the white matter at 8 days following LPS exposure (SAL vs 8 days LPS *p* = 0.004), in the hippocampus at 2, 4, 8, and 15 days following LPS exposure (SAL vs 2 days LPS *p* = 0.002; SAL vs 4 days LPS *p* = 0.030; SAL vs 8 days LPS *p* = 0.058; SAL vs 15 days LPS *p* = 0.042) and in the cortex at 8 days following LPS exposure (SAL vs 8 days LPS *p* = 0.041) compared to controls (Fig. 4a, b). No evidence of structural changes such as intraventricular hemorrhages and cystic lesions in all experimental groups was found. To assess the proliferation state of the brain, pHH3+ cells were counted. At 2 days following LPS exposure, a trend towards significant decrease in pHH3+ cells was found (SAL vs. 2 days LPS *p* = 0.100) (Fig. 4c).

### Distinct time-dependent changes in numbers of oligodendrocyte lineage cells following LPS exposure

**Fig. 5:**
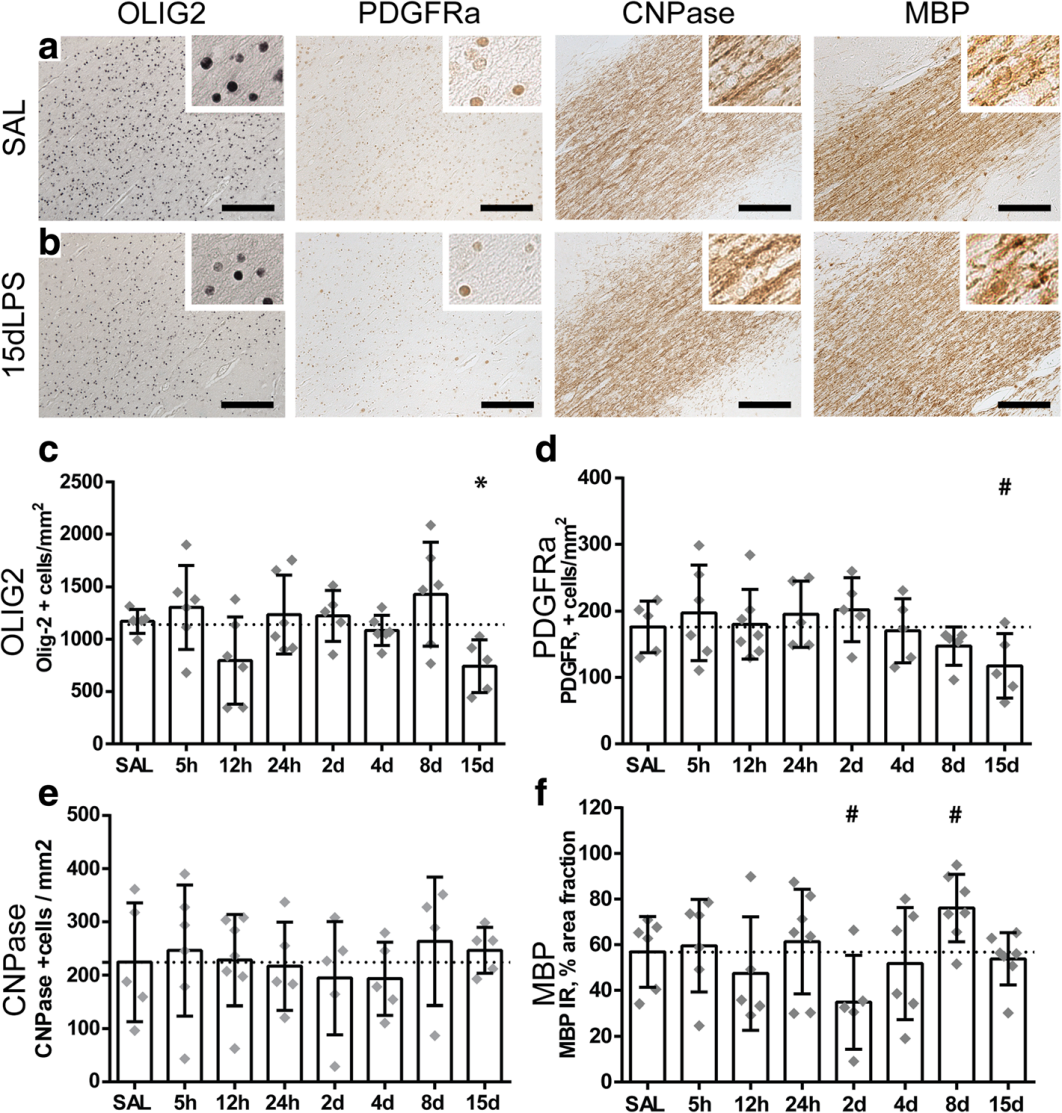
Intra-amniotic exposure to LPS induces distinct time-dependent changes in oligodendrocyte lineage cells. A significant decrease of Olig2-positive cells was observed in animals after 15 days of LPS exposure compared to controls (SAL vs. 15 days LPS *p* = 0.050) (**c**). At the same time point, a decrease in PDGFRa-positive cells was found compared to controls (SAL vs. 15 days LPS *p* = 0.070) (**d**). No significant changes of CNPase+ cells were found following LPS exposure compared to controls (**e**). Area fractions (%) of MBP immunoreactivity (IR) showed a decrease at 2 days and an increase at 8 days following LPS exposure compared to controls (SAL vs. 2 days LPS, *p* = 0.070; SAL vs. 8 days LPS *p* = 0.083) (**f**). Representative histological figures of Olig2, PDGFRa, and CNPase-positive cells and MBP IR in animals exposed to intra-amniotic saline (SAL) and 15 days of LPS are shown in **a** and **b** respectively. Images taken at × 100 magnification (insert at × 400 magnification), scale bar = 200 μm. Asterisk indicated *p* < 0.05 versus control (SAL); number sign indicated 0.05 < *p* < 0.1 versus control (SAL)

The typical histopathological substrate of brain injury in premature infants consists of injury of the developing oligodendrocyte (OL), a cell type that is abundantly present within the brain during weeks 23–32 of gestation (when preterm birth often occurs) and is prone to inflammatory insults [36]. Therefore, we have assessed oligodendrocyte development following intra-amniotic LPS exposure by studying the following oligodendrocyte lineage markers: Olig-2 as a pan-oligodendrocyte lineage marker, PDGFRα as a pre-oligodendrocyte marker (for both OL progenitor and pre-OLs), CNPase as an early oligodendrocyte differentiation marker, and MBP for mature oligodendrocytes and myelin. At 15 days after LPS exposure, a significant decrease in Olig2+ cell number was found compared to controls (SAL vs. 15 days LPS *p* = 0.050) (Fig. 5a, c). At this time point, the PDGFRa+ progenitor and precursor oligodendrocyte populations tended to decrease compared to controls (SAL vs. 15 days LPS *p* = 0.070) (Fig. 5a, d). No significant changes of CNPase+ cells and MBP IR were found following LPS exposure compared to controls at all studied time points (Fig. 5a, e, f).

### Intra-amniotic exposure to LPS resulted in altered dendritic development in gray matter regions of the fetal brain

**Fig. 6:**
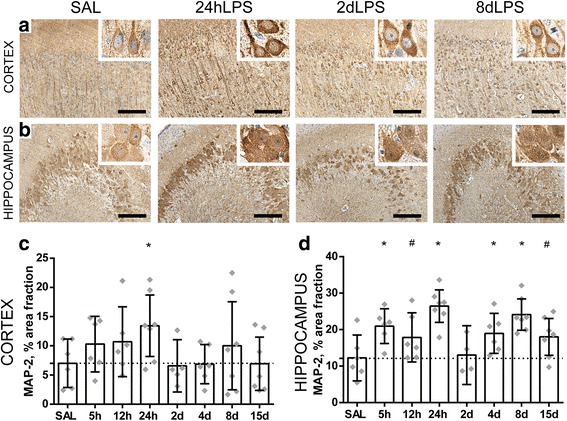
Intra-amniotic exposure to LPS results in altered dendritic development in the gray matter of the fetal brain. A significant increase of the area fraction (%) of MAP-2 immunoreactivity (IR) was found in the cerebral cortex at 24 h after LPS exposure compared to controls (SAL vs 24 h LPS *p* = 0.036) (**a**, **c**). In the hippocampus, an increase of area fraction (%) of MAP-2 IR was observed at 5, 12, and 24 h and at 4, 8, and 15 days after LPS exposure compared to controls (SAL vs 5 h LPS *p* = 0.007; SAL vs 12 h LPS *p* = 0.073; SAL vs 24 h LPS *p* = 0.000; SAL vs 4 days LPS *p* = 0.034; SAL vs 8 days LPS *p* = 0.000; SAL vs 15 days LPS *p* = 0.057) (**b**, **d**). Representative histological figures of MAP-2 in the cerebral cortex (**a**) and hippocampus (**b**) are depicted in control animals (SAL) and animals exposed to LPS for 24 h, 2 days, and 8 days. Images taken at × 100 magnification (insert at × 400 magnification), scale bar = 200 μm. Asterisk indicated *p* < 0.05 versus control (SAL); number sign indicated 0.05 < *p* < 0.1 versus control (SAL)

Besides alterations in white matter development and oligodendrocyte loss, developmental disturbances of the gray matter are an increasingly important feature of perinatal brain injury [37–39]. For the assessment of dendritic maturation, we have studied MAP-2 IR as an established marker for neuronal development [40] in the hippocampus and cerebral cortex. As illustrated in Fig. 5, intra-amniotic exposure to LPS results in a significant or trend to increase in the MAP-2 IR in the hippocampus at all time points except at 2 days LPS (SAL vs 5 h LPS *p* = 0.007; SAL vs 12 h LPS *p* = 0.073; SAL vs 24 h LPS *p* = 0.000; SAL vs 4 days LPS *p* = 0.034; SAL vs 8 days LPS *p* = 0.000; SAL vs 15 days LPS *p* = 0.057) (Fig. 6a, b). At 2 days following LPS exposure, MAP-2 IR is comparable to controls (SAL vs 2 days LPS *p* = 0.825). In the cerebral cortex only at 24 h after LPS exposure, an increase in MAP-2 IR was found (SAL vs 24 h LPS *p* = 0.036) (Fig. 6a, c) and all other time points were comparable to control.

### Expression of the phosphorylated erythropoietin receptor decreases 2 days following LPS exposure

**Fig. 7:**
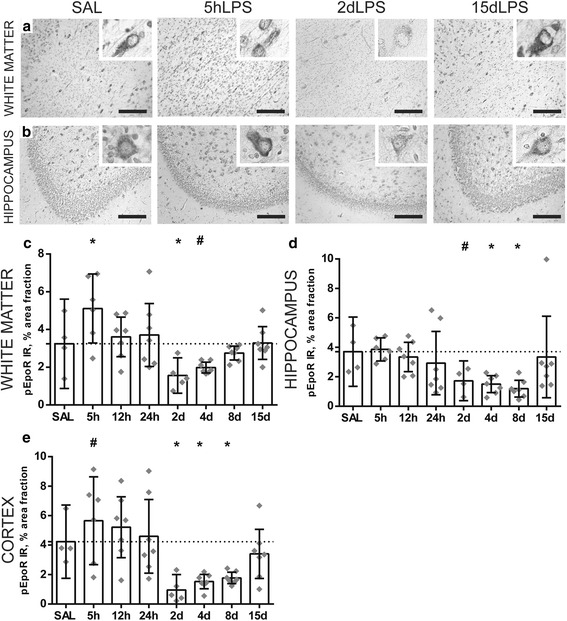
Expression of the phosphorylated erythropoietin receptor decreases 2 days following LPS exposure. An acute increase of the area fraction (%) of pEPOR immunoreactivity (IR) was observed at 5 h after LPS exposure in the white matter (SAL vs 5 h *p* = 0.010) and cortex (SAL vs 5 h *p* = 0.100) compared to controls (**c**, **e**). At 2 days after LPS exposure, there is a significant decrease in pEPOR IR within all brain regions compared to controls: white matter (SAL vs 2 days LPS *p* = 0.030) (**c**), hippocampus (SAL vs 2 days LPS *p* = 0.088) (**d**), and cortex (SAL vs 2 days LPS *p* = 0.010) (**e**). At 4 and 8 days following LPS exposure, pEPOR expression is still decreased compared to controls in the white matter (SAL vs 4 days LPS *p* = 0.100), hippocampus (SAL vs 4 days LPS 0.045; SAL vs 8 days LPS *p* = 0.014), and cortex (SAL vs 4 days LPS 0.020.; SAL vs 8 days LPS *p* = 0.030). When the fetus had been exposed to 15 days of LPS, there was no decrease in pEPOR IR (**c**–**e**). Representative histological figures of the pEPOR in the white matter (**a**) and hippocampus (**b**) are depicted in control animals (SAL) and animals exposed to LPS for 5 h, 2 days, and 15 days. Images taken at × 100 magnification (insert at × 400 magnification), scale bar = 200 μm. Asterisk indicated *p* < 0.05 versus controls (SAL), number sign indicated 0.05 < *p* < 0.1 versus controls (SAL)

There is a single EPO receptor and activation of this receptor leads to receptor phosphorylation and as such phosphorylation is a useful surrogate for EPO-induced downstream pathway activation. Analysis of the IR for total EPOR revealed no change from baseline levels at any of time points of LPS exposure. Interestingly, however, we observed that pEPOR had a distinct time-dependent switch from over-expression to under-expression. Specifically, following 5 h of LPS exposure pEPOR IR was significantly increased in the white matter (SAL vs 5 h *p* = 0.010) (Fig. 7a, c) and tended to increase in the cortex (SAL vs 5 h *p* = 0.100) (Fig. 7e) compared to controls. However, at 2 days after LPS exposure, there is a strong decrease in pEPOR IR within all brain regions compared to controls: significant in the white matter (SAL vs 2d LPS *p* = 0.030) and trending in the hippocampus (SAL vs 2 days LPS *p* = 0.088) and cortex (SAL vs 2 days LPS *p* = 0.010) (Fig. 7c–e). At 4 and 8 days following LPS exposure, pEPOR expression is still decreased compared to controls, trending in the white matter (SAL vs 4 days LPS *p* = 0.100), and significant in the hippocampus (SAL vs 4 days LPS 0.045; SAL vs 8 days LPS *p* = 0.014) and cortex (SAL vs 4 days LPS 0.020.; SAL vs 8 days LPS *p* = 0.030). When the fetus had been exposed to 15 days of LPS, there was no drop in pEPOR IR.

## Discussion

The main findings of this study are that intra-amniotic exposure to LPS results in (1) an acute onset and biphasic fetal systemic inflammatory response; (2) persisting microgliosis with a limited pro-inflammatory phase; (3) cell death and (pre) oligodendrocytes loss only following long exposure to inflammation (8 days +) but (4) a striking regional sensitivity to changes in neuronal architecture following short (hours) and long (days) LPS exposure, and (5) biphasic regulation of pEPOR expression. We demonstrate that fetuses, intra-amniotically exposed to LPS, develop biphasic opposing peaks in the systemic inflammatory response, illustrated by changes in IL-6 and IL-8 concentrations. This data agree with previous observations from this model on increased circulatory monocytes and neutrophils and subsequent lymphocytopenia [4] and agrees with previous clinical data showing increased levels of these cytokines, associated with adverse neurological outcomes [41, 9]. Furthermore, the systemic response to LPS is consistently associated with the production of a vast milieu of inflammatory cytokines including IL-6 [42]. These signals across the blood-brain barrier trigger a neuroinflammatory response including activation of microglia and astrocytes [6, 5]. Moreover, IL-8 is known as potent chemotactic and neutrophil-activating factor, and increased serum IL-8 levels are reported in newborns with MRI-defined cerebral abnormalities and abnormal neurodevelopmental outcomes [43, 44, 9, 45, 46]. In line, we observed an increased number of MPO+ cells within the cerebral white matter at 15 days after LPS exposure.

Our study also provides meaningful information about the cerebral inflammatory response initiated by intra-amniotic LPS exposure. We observed a rapid increase in the inflammatory mediator COX-2 and microglial marker IBA-1 in the preterm brain. COX-2 activity results in prostaglandin-E2 production which has wide ranging inflammatory actions on the brain [47], but is typically associated with a pro-inflammatory state in microglia [48] and also reported in astrocytes [49]. Weaver-Mikaere et al. demonstrated in a fetal ovine-derived mixed glial culture that COX-2 activation is the most important mechanism leading to inflammation-mediated white matter injury [50]. Blocking the COX-2 pathway has recently been shown to prevent hypomyelination and behavioral impairment in mice with neonatal white matter injury [49]. In this model, it has also been reported that there is also no change in GFAP-IR across the groups, but additional analysis revealed that despite this, astrocytes together with microglia were still an important source of oligodendrocyte-injurious COX-2 [49]. An additional injurious mechanism of increased COX-2 is that its primary product prostaglandinE2 stimulates glutamate release from astrocytes [51], which is suggested to be essential in the pathophysiological mechanism underlying neonatal encephalopathy [52]. Additionally, the dynamic temporal microglial response in our inflammatory model, indicated by increased IBA-1IR, is consistent with distinct phases of cerebral inflammation in response to other injurious factors in perinatal brain injury, including a hypoxic-ischemic or excitotoxic insult [9]. Microglial activation is proposed to be essential in cerebral injury and dysmaturation associated with exposure to maternal fetal infection/inflammation [53]. Of interest for the application of neurotherapeutics is that microglia activation based on the simple proxy of IBA-1IR was maintained for at least 8 days following LPS exposure, but increased COX2 as surrogate for a pro-inflammatory state was only elevated for 1 day. Further studies to determine a systemic surrogate of microglial activation state might re-invigorate the utility of previously discarded immunomodulatory neurotherapeutics if we understood when immunosuppression would be beneficial.

Considerable debate has raged in the field of preterm neuropathology regarding the role for cell death in encephalopathy of prematurity [54–57]. Experimental data has shown that microglia activation results in pro-inflammatory cytokine release which in turn can lead to apoptosis. However, experimental data has also demonstrated that moderate activation of microglia leads only to the maturation arrest of oligodendrocytes and not cell death [58]. Our study supports a coherent integration of clinical and experimental data as it shows that with a clinically relevant exposure paradigm, the duration of exposure is essential; oligodendrocyte death only occurs after 8 days of LPS exposure. Although outside the scope of this study, research into additional variables including pathogen type and maternal/fetal health and genetics also undoubtedly play a role understanding cohort-specific observations on neuropathology. We also wish to highlight that the significant loss of (pre) oligodendrocytes was not accompanied by myelin loss in our study. It has been previously postulated that the type of robust reduction in myelin protein levels that we would be able to measure with our analysis technique only occurs later in the course of brain injury due to normal kinetics of myelin production [59]. Furthermore, in an inflammation-induced oligodendrocyte injury model, early analysis of myelin proteins [58] and genes [60] has indeed failed to observe reductions despite later robust hypomyelination and behavioral deficits. Detailed analysis of myelin structure could be considered to investigate early markers of myelin injury in future studies.

Together with disturbed white matter development, alterations in gray matter development are implicated in long-term neurological sequelae following intra-amniotic infections and preterm birth [39, 37, 38]. During normal gray matter development, a decrease in MAP-2IR occurs which is indicative for refining of dendritic branching and spines [40]. Regulation of spine development is part of the basic homeostatic functions of microglia, which also include supporting immature cortical neuronal survival and stimulating myelination [61]. As such, increase in MAP-2 IR that we found in the acute phase following LPS exposure suggests a delay in cortical development, a phenomenon that has also been suggested from analysis of preterm infants using MRI [62]. Moreover, in humans suffering from schizophrenia, increased dendritic arborization was found in the hippocampus compared to healthy humans when stained for MAP-2 [63]. Concerning the specific mechanistic link between increased inflammation and increased MAP-2, it is noteworthy that COX-2 overexpressing neuronal-like cells showed significantly increased neurite outgrowth and branching, which was partially reverted by COX inhibitor [64].

Despite multiple studies identifying EPO as very promising neuroprotective candidate in newborn infants [65, 66, 14, 67, 13, 17, 68–70], other studies did not find therapeutic effects on neurodevelopmental outcomes [71, 72, 23]. These inconsistencies might be explained by heterogeneity between study cohorts including, as outlined in this study for the first time, differences in EPO signaling in response to inflammation. Interestingly, after an initial increase in the receptor phosphorylation levels, a distinct and long-lasting decrease of the pEPOR occurred. Activation of the EPOR phosphorylates activates the kinase Jak-2 resulting in initiation of a complex anti-apoptotic signaling cascade [73, 25]. Accordingly, we are unsurprised that a decrease in the level of pEPOR is accompanied by an increase in the number of apoptotic cells (caspase-3+) within the same region in the fetal brain. This suggests that the reduced activation of the EPO receptor in our study results in increased apoptosis in the preterm brain and in experimental paradigms this relationship between EPOR and cell death has been demonstrated [25]. In regards to the total expression of EPOR, others have also shown that this remains stable or increases [74], and this elevation occurs within hours in response to pro-inflammatory cytokines like TNFa [75], whereas the level of its parent ligand, EPO, is downregulated under pro-inflammatory conditions [76]. Thus, understanding the inflammation-induced dysregulation of EPO signaling provides a therapeutic window where treatment with EPO may be most efficacious [76]. Altogether, the considerable changes in EPOR expression within the fetal brain in the course of inflammation stresses the need for biomarkers to determine the onset of intra-amniotic infections.

One important limitation of a large animal study is the relatively low number of animals per group. Given the relatively small animal numbers per group, we reported actual *p* values and tended to interpret *p* values between 0.05 and 0.1 as biologically relevant. This assumption decreases the chance of a false-negative finding but increases the chance that one of these differences is a false-positive result.

## Conclusion

Altogether, the cerebral changes as found in our translational model of inflammation-related brain injury of the preterm infant could be reasonably expected to lead to deficits in learning, memory and social skills, and/or motor disabilities that can persist into adulthood [34]. However, these outcomes need to be confirmed in longitudinal studies, incorporating behavioral analysis and MRI but nonetheless inform the development of interventions to protect and/or regenerate the preterm brain in order to allow normal development. By gaining more insight into the temporal processes underlying inflammation-induced preterm brain injury, we provide an increased understanding of the pathophysiological changes in the fetal brain with the goal of helping to design new interventions or implement interventions more effectively to prevent perinatal brain damage.

## Abbreviations

ANOVA: Analysis of variance
CI: Confidence interval
CNPase: 2′,3′-Cyclic-nucleotide 3′-phosphodiesterase
COX-2: Cyclo-oxygenase 2
ELISA: Enzyme-linked immunosorbent assay
EPO: Erythropoietin
EPOR: Erythropoietin receptor
GFAP: Glial fibrillary acidic protein
H&E: Hematoxylin and eosin
IBA-1: Ionized calcium-binding adaptor molecule-1
IL-6: Interleukin-6
IL-8: Interleukin-8
IR: Immunoreactivity
LPS: Lipopolysaccharide
MAP2: Microtubule-associated protein 2
MBP: Myelin basic protein
MPO: Myeloperoxidase
Olig2: Oligodendrocyte transcription factor 2
PBS: Phosphate-buffered saline
PDGFRa: Platelet-derived growth factor receptor alpha
pEPOR: Phosphorylated erythropoietin receptor
pHH3: Phospho-Histone H3
SAL: Saline
SD: Standard deviation
TMB: 3,3′,5,5′-Tetramethylbenzidine
TNFa: Tumor necrosis factor alpha
